# Genome Analysis of a Marine Bacterium *Halomonas* sp. and Its Role in Nitrate Reduction under the Influence of Photoelectrons

**DOI:** 10.3390/microorganisms8101529

**Published:** 2020-10-05

**Authors:** Ying Liu, Hongrui Ding, Yuan Sun, Yan Li, Anhuai Lu

**Affiliations:** The Key Laboratory of Orogenic Belts and Crustal Evolution, Beijing Key Laboratory of Mineral Environmental Function, School of Earth and Space Sciences, Peking University, 100871 Beijing, China; liuy677@pku.edu.cn (Y.L.); sess_sy@pku.edu.cn (Y.S.); liyan-pku@pku.edu.cn (Y.L.)

**Keywords:** marine photic zone, photoelectron, nitrate reducer, draft genome

## Abstract

The solar light response and photoelectrons produced by widespread semiconducting mineral play important roles in biogeochemical cycles on Earth’s surface. To explore the potential influence of photoelectrons generated by semiconducting mineral particles on nitrate-reducing microorganisms in the photic zone, a marine heterotrophic denitrifier *Halomonas* sp. strain 3727 was isolated from seawater in the photic zone of the Yellow Sea, China. This strain was classified as a *Halomonadaceae*. Whole-genome analysis indicated that this strain possessed genes encoding the nitrogen metabolism, i.e., *nar*G, *nas*A, *nir*BD, *nor*Z, *nos*B, and *nxr*, which sustained dissimilatory nitrate reduction, assimilatory nitrate reduction, and nitrite oxidation. This strain also possessed genes related to carbon, sulfur, and other metabolisms, hinting at its substantial metabolic flexibility. A series of microcosm experiments in a simulative photoelectron system was conducted, and the results suggested that this bacterial strain could use simulated photoelectrons with different energy for nitrate reduction. Nitrite, as an intermediate product, was accumulated during the nitrate reduction with limited ammonia residue. The nitrite and ammonia productions differed with or without different energy electron supplies. Nitrite was the main product accounting for 30.03% to 68.40% of the total nitrogen in photoelectron supplement systems, and ammonia accounted for 3.77% to 8.52%. However, in open-circuit systems, nitrite and ammonia proportions were 26.77% and 11.17%, respectively, and nitrogen loss in the liquid was not observed. This study reveals that photoelectrons can serve as electron donors for nitrogen transformation mediated by *Halomonas* sp. strain 3727, which reveals an underlying impact on the nitrogen biogeochemical cycle in the marine photic zone.

## 1. Introduction

Estuary and offshore ecosystems are transitional zones between freshwater/terrene systems and marine environments, which are affected by both terrestrial and anthropogenic transportations [1]. Large amounts of suspended mineral particles are discharged from rivers, shore erosion by wave action, or resuspended from the bottom, resulting in elevated concentrations of minerals in coastal waters [2,3]. Mineral particles including semi-conductive minerals such as rutile, birnessite, and iron oxide have imperative effects on organisms in various ecological niches [4,5]. In the photic zone of the marine, through absorbing the solar energy, electrons in the valence band of semiconducting minerals transit to the conduction band, which has a higher energy level. This transition process provides a photoelectron in the conduction band and a photo-generated hole in the valence band. These photoelectrons can act as the electron donor for biotic metabolism and produce the origin organic matters on the earth [6,7], providing a substantial basis for the origin and evolution of marine life. Photoelectrons stimulating a pure culture of microorganism growth was proven with both natural and artificial semiconducting (nano)particles [8,9,10]. Meanwhile, in a complex microbial community, the influence of photoelectrons triggered by semiconducting minerals was also observed, including karst stones [11], red soils [12], and rock varnish [13]. Thus, the effects of photoelectrons generated by semiconducting minerals in the marine photic zone on microorganisms are worth studying in the natural environment.

Anthropogenic discharge of nutrients from agriculture, industries, sewage treatment, and septic tanks has led to a marked increase in the export of nutrients, particularly nitrogen, to nearshore coastal areas [14,15]. Increasing N loads (primarily as nitrate) associated with water quality problems in aquatic systems have received much attention [16,17]. Understanding the variables that affect the N cycle, and more specifically the pathways of nitrate reduction, has implications regarding the solution to those problems [17,18]. The ocean contains approximately five times more bioavailable N than the terrestrial system [19], and the transitional zones between freshwater and marine environments are critical [1,15]. Understanding the microbial-mediated nitrogen cycle also has implications for predicting the impact of excess nutrient inputs to nearshore marine systems [15,18], particularly based on the knowledge of denitrification processes [14,20]. Nitrate reduction is the first step of denitrification, i.e., dissimilatory nitrate reduction to ammonium and assimilatory nitrate reduction. Traditionally, denitrification has been studied in anaerobic environments [16,18]. With the discovery of aerobic denitrification [21], the roles of this process in various aerobic to micro-aerobic environments have received much attention [22,23].

This study, therefore, aimed to explore the effects of photoelectrons generated by semiconducting mineral particles on prokaryote-mediated nitrate reduction in the photic zone of the estuary area of the Yellow Sea. To elucidate the influence mechanism, marine nitrate reducers were isolated from seawater in the photic zone of the Yellow Sea. Genomic sequencing and statistical analysis were employed to determine the phylogenetic status and metabolic potential of the isolation. Because electrodes can serve as electron donors for denitrification under appropriate potential conditions [24], a series of microcosm experiments and a simulative photoelectron system were established to elucidate the underlying role of the isolated marine nitrate reducer in the denitrification process under different photoelectron conditions with different sources of electron donors. In the present study, several genes/enzymes identified in the genome were discussed, which might provide insights into its potential physiological roles. More importantly, the nitrate reduction pathways were verified with or without the influence of photoelectrons, which might help to clarify the influence of photoelectric effects of semiconducting minerals on the nitrogen metabolism of the isolate, which potentially shed light on the nitrogen biogeochemical cycle in the marine photic zone.

## 2. Methods and Materials

### 2.1. Enrichment and Isolation of Nitrate Reducer

**Figure 1:**
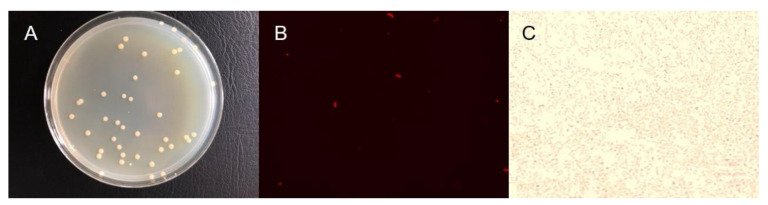
The colony of *Halomonas* sp. strain 3727 on LB solid medium (**A**); micrograph of cellular membrane staining using BBcellProbe^®^ membrane dye Dil (**B**); Gram staining micrograph (**C**).

The original prokaryote community of seawater in the Yellow Sea photic zone (33°–35°N, 120°–122°E) was enriched by a nitrate-reducing medium, which contained simulated seawater [25] amended with sodium nitrate (50 mg N/L) and acetate (0.51 g/L). The above liquid medium adding 5% agar was employed for the single-colony acquisition. The single colony was enriched and conserved temporarily in a LB solid medium (Coolabor, Beijing, China). The isolations were obtained with a single colony, which was of the same cell morphology characterized as ivory-white circular and smooth-edge (Figure 1A). The membrane dye Dil (Bestbio, Shanghai, China) was used to observe the morphology of the cells by a fluorescent microscope (Leica, Weztlar, Germany), and Gram staining was performed by a Gram staining kit (OKA, Beijing, China). The cell was rod-shaped with an average length of 3.48 ± 0.79 μm and was Gram-negative (Figure 1B,C). To determine the optimal pH for cell growth, the liquid LB medium was adjusted to different pH conditions (ranging from 6 to 10), and the cell growth was determined by OD_600_. The appropriate pH condition for cell growth was 7–9 (Figure S1).

### 2.2. DNA Extraction and Sequencing

The isolations were recovered and enriched in the LB liquid medium for 48 h under 35 °C. The total DNA of four colony isolations was extracted using ALFA-SEQ DNA Kit (mCHIP, Guangzhou, China) according to the manufacturer’s instructions. The 16S rRNA gene using primers 27F and 1492R was sequenced in Majorbio company (Shanghai, China) [26]. The16S rRNA gene sequences underwent BLAST analysis through the NCBI website to acquire the closest taxonomic status of the isolations. All four selected isolations had a 100% similarity, demonstrating that the colonies belonged to the same species with a >98% similarity to genus *Halomonas*. Thus, it was inferred that the four isolates were members of the same bacterial genus. Then, we select one isolation and named it as strain 3727. To predict the metabolic properties of strain 3727 and for assigning its correct phylogenetic position, we conducted whole-genome sequencing (WGS). The liquid culture using the medium for original isolation was used for obtaining a high concentration of biomass for DNA extraction that was used for genome sequencing. The sequencing was accomplished by the Hiseq X ten (Illumina, San Diego, CA, USA) in the Annoroad Genome company (Beijing, China).

### 2.3. Draft Genome Sequence and Phylogenetic Analysis of Strain 3727

Raw sequencing reads were filtered by Sickle (version 1.33). Then, high-quality datasets were assembled by SPAdes (version 3.11.0). The genome size was 5.12 Mbp, comprising 41 contigs with a total length of 5,388,179 bp. The average length of the contigs was 131,419 bp, and the average GC% was 54.69. The length of the longest contig was 850,481bp, and the N_50_ contig size was 295,618 bp. The average genomic coverage was approximately 2770-fold. The genome was then submitted to the JGI IMG/MER system for gene calling and annotation. A total of 4909 genes was predicted. Genes were annotated against the database of NCBI-NR, KEGG-Orthology (KO) (Table S1), eggNOG. For comparison, we also performed the same annotation analysis for the other two genomes (*H. titanicae* BH1 and *Halomonas* sp. UBA3171) that have close phylogenetic relations with our draft genome. Additionally, carbohydrate metabolism capacity was determined by the carbohydrate active enzymes (CAZymes) database, through CAZyme annotation (dbCAN) using a 1e^−6^ cutoff E-value and a 30% cutoff coverage value [27]. CAZymes are divided into five classes: glycoside hydrolases (GHs), glycosyl transferases (GTs), polysaccharide lyases (PLs), carbohydrate esterases (CEs) and auxiliary activities (AA) enzymes. The database also includes modules such as the cohesin module, dockerin, the S-layer homology (SLH) module, and carbon binding modules (CBMs). For Fe metabolism, a newly established FeGenie database that contains the genes for iron oxidation, iron reduction, iron transport, and regulation was used [28]. The genome was submitted to the NCBI SRA database, and the accession number for the genome is PRJNA628622.

The phylogenetic status of strain 3727 was determined by constructing a phylogenomic tree with 124 reference genomes, by using a concatenated alignment of 16 ribosomal proteins (16rp) as previously described [29]. The sequences of 16 ribosomal proteins of our draft genome and reference genomes were aligned by MAFFT (version 7.3.13) [30] and trimmed by TrimAL [31]. Then, the phylogenomic tree was built by IQ-TREE (version 1.6.10) [32].

### 2.4. Nitrate and Nitrite Reduction Supplemented with Sodium Acetate

Synthetic seawater supplemented with potassium nitrate (ca. 30 mg N/L) or sodium nitrite (ca. 10 mg N/L) and sodium acetate (C/N = 5) was injected into a 100 mL serum bottle and sealed with a butyl rubber stopper and aluminum crimp cap. Triplicates were set for each experimental setting. Then, sterilization was carried out by autoclave under 121 °C for 23 min. Strain 3727 was recovered in LB liquid medium for 48 h, and 10 mL culture medium was extracted and centrifuged for 10 min under 4000 rpm. Afterward, it was resuspended and washed by 20 mL 0.9% NaCl thrice and then resuspended by 5 mL synthetic seawater before being injected into the serum bottle. All serum bottles were cultured in an incubator under 35 °C 150 rpm. Samples were taken at days 0, 2, 6, 8, 9, and 11.

### 2.5. Photoelectron System Setup

The simulative photoelectron system was established by a dual-chamber electrochemical reactor with a volume of 120 mL in both cathode and anode chambers, as a previous study described (Liu et al., 2020b). The two chambers were separated by a proton exchange membrane (PEM, Dupont, USA). Sterile synthetic seawater and normal saline washed cells of strain 3727 (same as above) were injected into the cathode and anode, respectively. A pectinate and tabular graphite electrode, for the cathode and anode acting as working and counter electrodes, was pretreated in 1 mol/L HCl for 1 h, cleaned by deionized water, and soaked in 1 mol/L NaOH for 1 h. After ultrasonic treatment in deionized water for 10 min, the electrodes were ready for use. A saturated calomel electrode (SCE, 0.244 V vs. normal hydrogen electrode) served as the reference electrode. Reactors were set with open circuit (OC), low (L; -0.15 V vs. SCE), medium (M; -0.20 V vs. SCE), and high (H; -0.30 V vs. SCE) (triplicates were operated under each condition) potentials maintained by an electrochemical workstation (CHI 1000C Shanghai Chenhua Instrument, China) (Table 1).

### 2.6. Chemical Analysis Methods

For each experiment, 3 mL samples were taken six times during the experiments and filtered by a 0.22 μm filter membrane (Jinteng, China). The concentrations of nitrate, nitrite, and ammonia were measured by standard methods as previously described [33]. The pH value was measured by a pH meter (FE20, Mettler Toledo, Switzerland). The current was auto-recorded by the electrochemical workstation, and the potential between the working electrode and the counter electrode was measured by an Avometer every day.

## 3. Results and Discussion

### 3.1. Genome-Resolved Metagenomic and Phylogenetic Analyses

**Figure 2:**
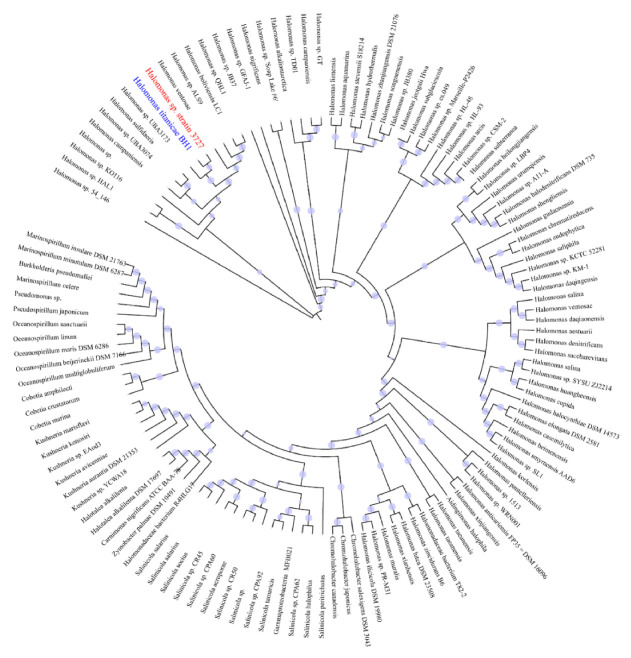
Phylogenetic placement of *Halomonas* sp. strain 3727. The maximum-likelihood phylogenetic tree was constructed using the concatenated alignment of 16 ribosomal proteins. Bootstrap values were based on 100 replicates, and percentages ≥50% are shown with light blue circles.

The phylogenomic tree of the isolated strain obtained from WGS was constructed (Figure 2). Strain 3727 was located at a position between its marine relatives *H. titanicae* BH1 and *Halomonas* sp. UBA3171. This result was consistent with the phylogenetic position of strain 3727 inferred by the 16S rRNA gene sequence. Additionally, the orthologous average nucleotide identity (orthoANI) was calculated among the draft genome of strain 3727, *H. titanicae* BH1, and *Halomonas* UBA3171. The orthoANI values between strain 3727 and *Halomonas titanicae* BH1 or strain 3727 and *Halomonas* sp. UBA3171 were 99.65% and 94.20%, respectively. The genus *Halomonas* belongs to the *Halomonadacea* family and is distributed widely in environments with high salinity, i.e., saline-alkali soil, salt lakes, and marine environments [34]. The first species of the *Halomonas* genus was the typical strain *Halomonas elongate* reported by Vreeland et al. [35]. The members of *Halomonas* are halotolerant or strictly halophilic, and most of them are capable of tolerating a wide range of pH values [34]. Because the genomes with ANI higher than 97% were used to classify the genomes at a species level [36], we inferred that our isolated bacterium strain 3727 was affiliated with *Halomonas titanicae*. *Halomonas titanicae* was first isolated from the surface of the wreckage of the Titanic [37]. Despite the high ANI between strain 3727 and *Halomonas titanicae* BH1, the different biotopes could induce potentially different metabolic characteristics.

### 3.2. Draft Genome Features of Strain 3727

#### 3.2.1. Carbon Metabolism

**Figure 3:**
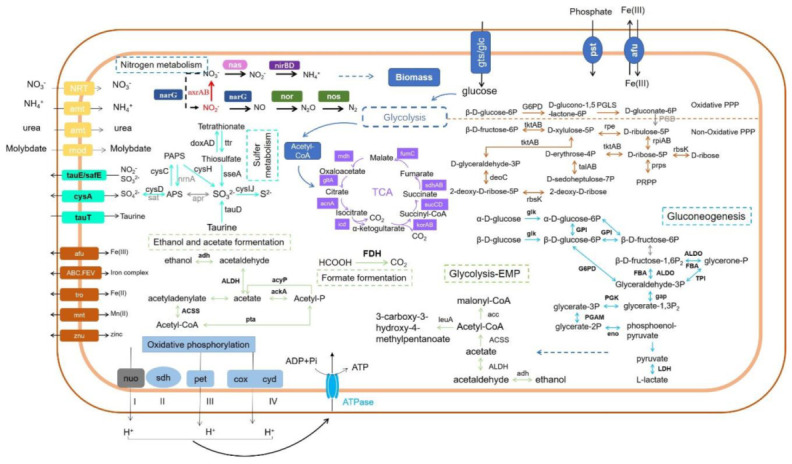
Metabolic capabilities of *Halomonas* sp. strain 3727. Pathways associated with carbon, nitrogen, and sulfur are shown, along with oxidative phosphorylation, fermentation, and ion transportation.

From the genomic information, strain 3727 is a putative chemoheterotrophic bacterium, as no genes involved in the carbon fixation pathway were found, but genes involved in some organic carbon metabolisms such as glycolysis, the tricarboxylic acid (TCA) cycle, the pentose phosphorylation pathway (PPP), and gluconeogenesis were identified (Table S1, Figure 3). The presence of these genes is suggestive of substantial carbon metabolic flexibility in this microbe. For instance, genome annotation suggested that this strain also harbored genes for the oxidation of ethanol, acetate, and formate via gene *adh*, ALDH, *ack*, FDH, and transformed to acetyl-CoA, which further entered into other pathways like the tricarboxylic acid cycle or oxidized to carbon dioxide through fermentation, glycolysis, and TCA. Although the strain possessed lactose and fructose assimilation genes, which are found in other species of *Halomonas* such as *Halomonas* sp. SF2003 [38], *H. marina*, and *H. halodurans* [39], due to the infrequency of multiple sugars in marine environments, whether this species has these functions is currently unclear. The annotation result via the CAZy database revealed that strain 3727 contains genes coding for GHs, GTs, CEs, CBMs, and AAs (Table S3). The results indicated that this strain might have the potential to metabolize various organic compounds.

#### 3.2.2. Nitrogen Metabolism

The nitrogen cycle is ubiquitous and active in the marine environment [40]. A set of genes required in performing denitrification was found in this species, including membrane-associated nitrate reductase gene *nar*G, nitric oxide reductase gene *nor*BC, and nitrous oxide reductase *nos*Z, but no nitrite reduction genes (either *nir*K or *nir*S) were detected (Figure 3). The gene encoding periplasmic nitrate reductase *nap*A was not identified in the genome. The genes for dissimilatory nitrate reduction to ammonia, i.e., *nar*G and *nir*BD, were detected in the genome. For assimilatory nitrate reduction, nitrate reductase *nas*A was identified, but the genes encoding assimilatory nitrite reduction to ammonia (*NIT-6* and *nir*A) were not identified in the genome. Besides, nitrite oxidase gene *nxr*AB was also identified. No genes related to nitrogen fixation and ammonia oxidation were found. The inorganic nitrogen compounds acquisition and regulator genes including *NRT*, *amt* (also for organic urea), and *tau*E/*saf*E were also found. The results together suggest that strain 3727 might be involved in the marine nitrogen biogeochemical cycle via reducing nitrate to gas dinitrogen and ammonia.

#### 3.2.3. Sulfur Metabolism

The genes encoding sulfur metabolism were identified in the genome of strain 3727 (Figure 3). Genome annotation suggested that genes encoding assimilatory sulfate reduction (*cys*D, *cys*C, *cys*H, and *cys*IJ) via reduction of sulfate to sulfide were identified in strain 3727. The genes *dox*AD (thiosulfate dehydrogenase) or *ttr* (tetrathionate reductase) were also found, illustrating the potential for tetrathionate reduction to thiosulfate or the reverse process. Then, thiosulfate might be reduced to sulfite (by thiosulfate/3-mercaptopyruvate sulfur transferase). The genes encoding taurine reductase (*tau*D), which mediated taurine reduction to sulfite, and sulfite reductase catalyzing (*cys*IJ) sulfite to sulfide were also identified in the genome. These results suggested that strain 3727 may have the potential to be involved in the marine sulfur cycle.

#### 3.2.4. Other Metabolisms

The genome possessed a series of genes encoding subunits of the electron transport chain for oxidative phosphorylation (Table S1, Figure 3). For instance, *Halomonas* sp. strain 3727 harbored the gene sets of Complex II (succinate dehydrogenase, *sdh*) and ComplexIII (ubiquinone-cytochrome *c* reductase, *pet*). Furthermore, genes encoding various types of terminal oxidases, such as cytochrome c-type *aa_3_* (*cox*), cytochrome bd (*cyd*), and quinol oxidase, were present. Previous studies demonstrate that this system can execute electron transfer and energy production [41].

Iron is one of the most important elements for microorganisms, as iron constitutes the reactive center of various enzymes [42,43]. The iron-related gene was annotated (Table S2). The genes involved in the iron acquisition, regulator, and transporter were identified. For instance, in the category iron acquisition, functions such as siderophore transport (such as ExbB/D family, FpvE family, LucA family), siderophore synthesis (PvdA/E/F-family, LucB-family, VabF family, PchC-family), heme transport (HmuV family), and iron transport (FbpB-FutB-family, FutA2-family, YfeA/YfeB-family) were found. Genes related to iron regulation and storage belong to FecR and Ftn families.

Past studies suggested that the salinity adaptation of some species of *Halomonas,* such as *Halomonas* sp. R5–57 and *H. elongate* DSM 2581, was due to the presence of an osmoprotectant: the compatible solute 1,4,5,6-tetrahydro-2-methyl-4-pyrimidinecarboxylic acid, namely ectoine [44,45]. Genes encoding the synthesis of this cyclic amine include *ect*A, *ect*B, and *ect*C. Gene *ect*D was responsible for ectoine hydroxylase, which induced ectoine derivative synthesis [38,44]. In this case, from a genomic perspective, these genes were also identified in the genome of *Halomonas* sp. strain 3727, which was isolated from a saline environment. To be noted, the identification of these genes is not able to directly infer the real function of this microbe, and further efforts are required to verify these pathways.

#### 3.2.5. Comparison of Strain 3727, *Halomonas titanicae* BH1, and *Halomonas* UBA3171

Because strain 3727 shares a high similarity with the genomes BH1, the genomic information is similar between these two species. In contrast, differences in genomes of strain 3727 and UBA3171 were observed (Table S1). As compared to strain 3727, the genome of *Halomonas* UBA3171 lacks genes encoding tetrathionate reductase (*ttr*ABC), Mn (II)/Fe(II) transporters (*mnt*H), and iron complex outer membrane receptor protein. Conversely, *Halomonas* UBA3171 has a cobalt-zinc-cadmium efflux system protein (*czc*D) and pyruvate dehydrogenase E2 component (dihydrolipoamide acetyltransferase) (DLAT), which are not found in the genome of strain 3727. The difference in these functional genes raises the potential that these two species may have different sulfur metabolisms and tolerances to metal ions in the marine environment.

### 3.3. Nitrogen Transformation Potential of Halomonas Strain 3727 under Photoelectron Impact

Because the nitrogen cycle causes nitrogen sink and loss in the marine environment, we established a series of microcosm experiments to test the capability of strain 3727 for nitrate and nitrite transformations. Both nitrate reduction and nitrite reduction were conducted using acetate as an electron donor. For the nitrate reduction experiment, nitrate reduction efficiency was 54.32% ± 9.54%. Nitrate was mainly reduced to nitrite, and ammonia was under the detected limit. For the nitrite reduction experiment, the nitrite reducing efficiency was 65.43% ± 14.96%, of which ca. 24.86% nitrite was oxidized to nitrate, and ammonia was also not detected during the experimental process. Thus, strain 3727 proved to be capable of nitrate and nitrite reduction using labile organic carbon as the electron donor.

**Figure 4:**
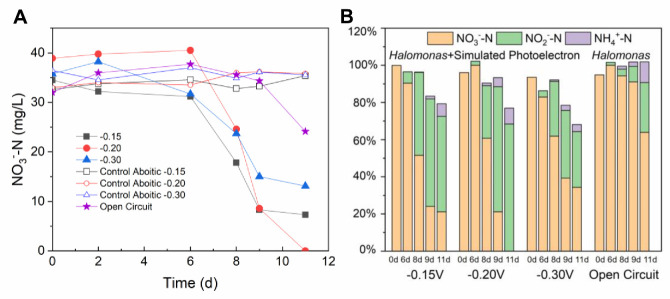
Time-course change of nitrate concentrations (**A**) and percentage of different N-compounds (nitrate, nitrite, ammonia) (**B**) in different experimental settings.

In addition to organic carbon, the photoelectrons generated by semiconducting minerals were proven to sustain the metabolism of microorganism and had an influence on microbial ecology on terrestrial environments [11,12,46]; thus, their role in the nitrogen cycle in the photic zone of marine areas was worth exploring. The dual-chamber electrochemical system was built for providing different energy-containing simulated photoelectrons for the nitrate reduction of strain 3727. The nitrate concentration of abiotic controls was stable during the experiment, indicating that the nitrate could not be reduced directly by electrode electrons (Figure 4). With different energy-containing simulated photoelectrons, nitrate reduction efficiencies reached 78.80% (−0.15V), 100% (−0.20V), and 59.26% (−0.30V), which was much higher than that without supplying simulated photoelectrons (36.06% in the open-circuit control). The first-order reaction constants were 0.29 d^−1^, 0.74 d^−1^, 0.18 d^−1^, and 0.09 d^−1^ in experimental settings for −0.15, −0.20, −0.30 V and OC, respectively. The highest reaction constant in the condition of -0.20 V was 8.2-fold higher than that in OC. This result indicated that simulated photoelectrons could increase the efficiency and rate of nitrate reduction mediated by *Halomonas* sp. strain 3727. The nitrate concentration in OC did not display an obvious decreasing trend until day 9, suggesting no nitrate reduction. In contrast, because of the presence of simulated photoelectrons, the nitrate had been reduced in other experimental settings before day 9. The final products of nitrate reduction retained in the solution included nitrite and ammonia (Figure S2). In the systems with supplemented photoelectrons, the by-product nitrite, which was the main product of nitrate reduction, accounted for 30.03%–68.40% of the total nitrogen, and ammonia accounted for 3.77%–8.52% of the total nitrogen (Table 1). In the OC system, nitrite accounted for 26.77% of the total nitrogen, ammonia accounted for 11.17%, and nitrogen loss in the liquid phase was not observed. This result demonstrated different end N-compound component distributions of nitrate reduction under different electron sources and electron energy (Figure 4B).

The functional genes should be responsible for the above observations of the nitrate reduction process. The dedicated nitrite reductase genes, i.e., *nir*K (copper-containing nitrite reductase, CuNiR) and *nir*S (cytochrome d_1_-containing nitrite reductase, Cd_1_NiR), were not found in the genome of strain 3727. However, nitrite was reduced in our experiments; thereby, the “no-dedicated” nitrite reductase was considered for nitrite reduction. The “no-dedicated” nitrite reductase included hemic globin, aldehyde oxidoreductases (AOR), and membrane-bound nitrate reductase (NarGHI), which contain a molybdenum center [47]. In strain 3727, membrane-bound nitrate reductase (NarG) was assumed to be responsible for both nitrate and nitrite reduction. This reaction calls for nitrate sufficiency and nitrite-accumulating conditions, which would induce the enzyme expression and promote the reaction [48]. This is the reason that in the nitrite reduction system nitrite was oxidized to nitrate as a result of NarG gene expression. Nitrate would competitively inhibit nitrite reduction, while promoting nitrite reduction when the nitrate concentration decreased and nitrite concentration increased [48,49].

In the photic zone of marine environments, the light-motivated photoelectrons generated by semiconducting mineral particles might play an imperative role as electron donors for prokaryote metabolism [46]. The present study illustrated that *Halomonas* strain 3727 can use photoelectrons for self-metabolism. The photoelectrons with different energy had various effects on the nitrate reduction kinetics and product distribution mediated by this species. Although different effects of electrons with different energy were observed on nitrate reduction by *Halomonas* sp. strain 3727, the influence of photoelectrons on basic metabolism and gene transcription is also worth exploring in future studies.

## 4. Conclusions

A nitrate reducer was obtained from the seawater of the photic zone of the Yellow Sea. Through whole-genome and phylogenomic analyses, the isolate was classified to genus *Halomonas* and named as strain 3727, which is a putative chemoheterotroph with potentials involved in versatile metabolisms, including nitrogen and other metabolic metabolisms. Combined with laboratory experiments and identified nitrogen metabolism genes, we suggest that strain 3727 can perform dissimilatory and assimilatory nitrate reduction and nitrite oxidation, and the nitrate and nitrite reduction might be catabolized by membrane-bound nitrate reductase, NarG. Simulated photoelectrons could sustain the nitrate reduction by strain 3727 under different electron energy situations, and the reaction kinetics and products differed from one to another. This study introduces a concise sight of photoelectron-fed nitrate reduction by a prokaryote in the aerobic photic zone of the marine environment, which may help to better understand the marine nitrogen biogeochemical cycle.

## Figures and Tables

**Table 1:** Inorganic N compound distribution, Faradaic efficiency and electrons accepted by N compounds.

Potential	NO_3_^−^-N	NO_2_^−^-N	NH_4_^+^-N	N Loss in Liquid Phase	Faradaic Efficiency
−0.15V	21.20%	51.36%	6.77%	20.67%	25.28%
−0.20V	0.00%	68.40%	8.52%	23.08%	4.28%
−0.30V	40.74%	30.03%	3.77%	25.19%	3.82%
Open Circuit	63.94%	26.77%	11.17%	0.00%	/

N_2_ was assumed to be the product for N loss in calculating Faradaic efficiency.

## Supplementary Materials

The following are available online at https://www.mdpi.com/2076-2607/8/10/1529/s1, Figure S1: Growth of *Halomonas* sp. strain 3727 under different pH conditions, Figure S2: Ternary phase diagram of N compounds component distribution in liquid phase at the end of the experiments, Table S1: The number of genes assigned to metabolic pathways, Table S2 Identification of genes involved in iron oxidation, dissimilatory iron reduction, and possible iron oxidation and reduction, Table S3 Identification of genes involved in CAZy.
