# Supplementary material for: Genome Analysis of a Marine Bacterium Halomonas sp. and Its Role in Nitrate Reduction under the Influence of Photoelectrons
Source: Microorganisms. 2020 Oct 5;8(10):1529. doi: 10.3390/microorganisms8101529 (PMC7650824; doi:10.3390/microorganisms8101529)
Supplement: Supplementary file 1 [file microorganisms-08-01529-s001.zip › supporting information.docx]

**Genome analysis of a marine bacterium *Halomonas* sp. and its role in nitrate reduction under the influence of photoelectrons**

Ying Liu, Hongrui Ding*, Yuan Sun, Yan Li, Anhuai Lu*

*The Key Laboratory of Orogenic Belts and Crustal Evolution, Beijing Key Laboratory of Mineral Environmental Function, School of Earth and Space Sciences, Peking University, Beijing, China*

**^*^Correspondence:**

Dr Anhuai Lu

School of Earth and Space Sciences, Peking University, Beijing, China

Email: [ahlu@pku.edu.cn](mailto:ahlu@pku.edu.cn)

Dr Hongrui Ding

School of Earth and Space Sciences, Peking University, Beijing, China

Email: [dhr_100@163.com](mailto:dhr_100@163.com)





Figure S1 Growth of *Halomonas* sp. strain 3727 under different pH conditions.


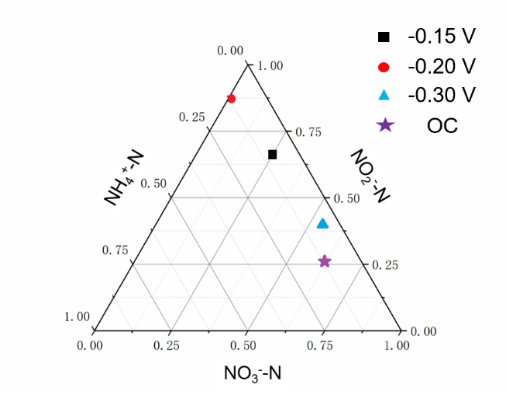


Figure S2 Ternary phase diagram of N compounds component distribution in liquid phase at the end of the experiments.
